# Influence of Parasite Load on Renal Function in Mice Acutely Infected with *Trypanosoma cruzi*

**DOI:** 10.1371/journal.pone.0071772

**Published:** 2013-08-12

**Authors:** Juliana Regina Dias Lemos, Wellington Francisco Rodrigues, Camila Botelho Miguel, Ricardo Cambraia Parreira, Renata Botelho Miguel, Alexandre de Paula Rogerio, Carlo Jose Freire Oliveira, Javier Emilio Lazo Chica

**Affiliations:** 1 Postgraduate Course of Pathology, Federal University of Triângulo Mineiro, Uberaba, Minas Gerais, Brazil; 2 Institute of Biological and Natural Sciences, Federal University of Triângulo Mineiro, Uberaba, Minas Gerais, Brazil; 3 Department Nutrition and Dietetics, Portuguese Beneficent Hospital of Uberaba, Uberaba, Minas Gerais, Brazil; 4 Laboratory of Experimental Immunopharmacology, Federal University of Triângulo Mineiro, Uberaba, Minas Gerais, Brazil; 5 Postgraduate Course of Tropical Medicine, Federal University of Triângulo Mineiro, Uberaba, Minas Gerais, Brazil; Federal University of São Paulo, Brazil

## Abstract

**Background:**

Chagas disease is a neglected tropical disease caused by *Trypanosoma cruzi*. Despite the vast number of studies evaluating the pathophysiological mechanisms of the disease, the influence of parasite burden on kidney lesions remains unclear. Thus, the main goal of this work was to evaluate the effect of *T. cruzi* infection on renal function and determine whether there was a correlation between parasite load and renal injury using an acute experimental model of the disease.

**Methodology/Principal Findings:**

Low, medium and high parasite loads were generated by infecting C57BL/6 mice with 300 (low), 3,000 (medium) or 30,000 (high) numbers of “Y” strain trypomastigotes. We found that mice infected with *T. cruzi* trypomastigotes show increased renal injury. The infection resulted in reduced urinary excretion and creatinine clearance. We also observed a marked elevation in the ratio of urine volume to kidney and body weight, blood urea nitrogen, chloride ion, nitric oxide, pro- and anti-inflammatory cytokines and the number of leukocytes in the blood and/or renal tissues of infected mice. Additionally, we observed the presence of the parasite in the cortical/medullary and peri-renal region, an increase of inflammatory infiltrate and of vascular permeability of the kidney. Overall, most renal changes occurred mainly in animals infected with high parasitic loads.

**Conclusions/Significance:**

These data demonstrate that *T. cruzi* impairs kidney function, and this impairment is more evident in mice infected with high parasitic loads. Moreover, these data suggest that, in addition to the extensively studied cardiovascular effects, renal injury should be regarded as an important indicator for better understanding the pan-infectivity of the parasite and consequently for understanding the disease in experimental models.

## Introduction

The kidneys are responsible for a diverse range of functions including homeostatic, regulatory and excretory functions as well as hormone production. Injury of the kidneys may be associated with complications such as anemia, increased blood pressure, decreased quality of life or even death. Infectious diseases caused by pathogens such as viruses, bacteria and protozoa may affect the normal function of the kidneys [1]–[3]. Chagas disease, discovered by Carlos Chagas in 1909 [4], is a severe American parasitosis caused by the hemoflagellate protozoan *Trypanosoma cruzi*. This disease is a major public health concern in Latin America, where it is estimated to affect 12–14 million people [5]. In non-endemic countries, Chagas disease has also been reported as consequence mainly of the migration of infected people from endemic countries (Latin America) to these areas [6]. Additionally, this disease causes the death of approximately 21,000 people each year worldwide [7].

Clinically, Chagas disease usually develops from an acute to a possibly debilitating chronic phase. Although the majority of patients remain clinically asymptomatic for many years, the chronic phase of the disease can include cardiac, digestive, cardiac/digestive or nervous system manifestations [8], [9]. The acute phase of the disease has been extensively investigated in the clinical and experimental settings, and the symptoms may include fever, myalgia, malaise, hepatosplenomegaly, acute robust myocarditis and innate and acquired immune changes [10], [11]. The involvement of the kidneys in the acute phase of the disease remains poorly described, despite the ability of *T. cruzi* to parasitize a wide variety of host cells, including renal cells [12].

Most of the publications focusing on Chagas disease and kidney function are associated with organ transplantation [13]–[15]. Nevertheless, there is some evidence of structural and functional changes in the kidney after *T. cruzi* infection. BALB/c mice infected with the “Y” strain presented renal lesions related to decrease of blood flow and injury in the proximal renal tubules at 6 days post-infection [16]. It was also demonstrated that the absence of Fas-L, a type-II transmembrane protein involved in apoptosis, intensely aggravated renal injury in acute *T. cruzi* infection [17]. Despite these studies, the relationship between *T. cruzi* and kidney injury, as well as the nature of the histopathological, immunological and functional alterations, remains unclear. Moreover, although some published works suggest that the number of parasites present influences the development of chronic Chagas disease pathology in different organs [18]–[21], no studies have evaluated the effect of parasite burden on kidney injury. Thus, the aim of this study was to describe the histopathological, immunological and functional alterations in the kidney during the acute phase of Chagas disease in mice infected with different parasite loads.

## Methods

### Animals

Male C57BL/6 mice weighing 20–30 g (6–8 weeks old) were housed in temperature-controlled rooms (22–25°C) with access to water and food *ad libitum*. All experiments were performed in accordance with the National Health guidelines for the welfare of experimental animals and with the approval of the Ethical Committee of the University Federal of Triângulo Mineiro (process number: 150/2010). None of the animals were used in more than one experimental group. The animals were divided into the following groups: uninfected, infected with 3×10^2^ (low), 3×10^3^ (medium) or 10^4^ (high) trypomastigotes.

### Parasite Strain and Mouse Infection

Mice (10 animals per group) were infected by subcutaneous injection of the blood-derived “Y” strain of trypomastigotes (MHOM/BR/00Y; T. cruzi lI) [22]–[24], which was kindly provided by the University of São Paulo (Brazil) and maintained in the Department of Cell Biology at Federal University of Triângulo Mineiro (Uberaba, Brazil).

### Parasitemia and Survival

Parasitemia was measured by the method of Brener [25]. Parasites were counted in 50 microscopic fields of a wet preparation that contained 5 µl tail blood under a 22 x 22 mm coverslip. The parasitemia count was performed every 3 days until the thirtieth day of infection. The results were expressed as parasites/mL. In other experiments, mice were infected with 3×10^2^, 3×10^3^ or 3×10^4^ trypomastigotes and the mouse survival rate was recorded daily.

### Biological Samples

Based on the parasitemia curve, the biological samples, except for urine, were collected at 6, 9, 12 and 18 days post-infection. Urine samples were collected at the day before euthanasia over a period of 24 hours with the use of metabolic cages and were then centrifuged at 1831 x *g* for 10 minutes and frozen (−20°C) until used for biochemical tests. We also measured the length and body weight of the animals in the different groups. After fasting for 6 hours, the animals were heparinized and euthanized in a CO_2_ chamber. The blood was then drawn through the ophthalmic plexus, centrifuged at 1831 x *g* for 10 min to obtain the plasma and stored at −70°C until used for biochemical tests. A closing-pubic incision was used to open the thoracic and abdominal cavities to collect the kidneys.

### Correlation between Urine Volume (mL per 24 Hours) and the Kidney to Body Weight Ratio

The kidney weight (KW) and body weight (BW) of each animal was measured at each time point, and the relationship between them was calculated (KW/BW). Subsequently, we calculated the correlation between the volume of urine excreted (mL/24 hours) and the KW/BW ratio.

### Creatinine Clearance (CrCl)

Plasma and urinary creatinine (in urine/24 hours) were quantified using commercial kits from Biotechnical® (Ref: 10.007.00) that use a kinetic (2 points) colorimetric method (red-yellow) based on picrate in an alkaline solution. Absorbance readings were performed using a semi-automated method in a spectrophotometer (Bioplus ® −2000) at a wavelength of 500 nm. We used the weight and length of each animal to calculate the median body surface area (XMBS) with the following equation: XMBS = ΣBS/N, where N = total number of animals and BS = (weight (W)^ 0.425^ x length (L)^ 0.007184^. The CrCl was expressed in mL/min and was obtained using the following equation: clearance (mL/min) x (XMBS)/BS, where the clearance was equal to the concentration of the urine creatinine (mg/dL) divided by the concentration of the plasma creatinine (mg/dL) and multiplied by the urinary volume over 24 hours (mL).

### Urea

To obtain the levels of plasma urea, we used a commercial kit from Biotechnical® (Urea UV - Ref: 10.012.00). This kit uses kinetic reaction with absorbance measured at two time points using a wavelength of 340 nm. After determining the urea concentrations, the values for blood urea nitrogen (BUN) were calculated using the following calculation: BUN = urea x 0.46.

### Chlorine

Plasma chlorine was measured using a commercial Biotechnical® (Ref: 12.003.00) kit, and the values were expressed as mEq/L. The chloride ions in the plasma react with mercuric thiocyanate to form chloride mercury and thiocyanate ions that then react with ferric ions to form the red compound ferric thiocyanate. The amount of ferric thiocyanate was proportional to the concentration of chloride in the sample and can be measured at a wavelength of 500 nm.

### Quality Control

We performed an internal quality control where all of the following parameters were upheld: clear definition of objectives, procedures, standards and criteria for the tolerance limits, corrective actions and registration of the activities and the use of controls to evaluate the imprecision of the analysis, applying the Westgard Rules [26].

### Histological and Immunohistochemical Analysis

For histological processing, the kidneys were placed in methacarn for 30 minutes and then stored in 70% alcohol until they were used. Kidneys were processed using dehydration, inclusion and diaphanization followed by microtomy. The blocks were cut in a rotary microtome to obtain sections of five micrometers. The sections were put on slides (five slides per kidney), and the procedure was repeated until all the slides contained four sections each. This procedure was performed ten times without discarding any slices. To evaluate the structural morphology of the kidney, the glomerular volume was calculated. This volume corresponds to the product of the numerical density (in mm^3^) divided by the volume of the kidney (Vk). To determine the numerical density for each animal, we analyzed two sections, and the counts for the number of glomeruli in a known area from each section (test system) were recorded. Knowing the distance between the two cuts and the depths at which the cuts were made, we were able to calculate the volume of the kidney between the two cuts. By dividing the number of glomeruli found in the test system by the volume, we determined the numerical density. The renal volume corresponds to the product of the kidney weight multiplied by 1.0048 [27].

Additional sections were mounted on glass slides and were used for immunohistochemical analyses. The slides were pre-treated with 3-aminopropyltriethoxy-silane (Sigma, St. Louis, MO, USA), immersed in xylene for 10 min to eliminate the paraffin, dehydrated in absolute alcohol and re-hydrated with Tris-buffered saline (TBS). The sections were rinsed in TBS and immersed in a 3% hydrogen peroxide methanol solution for 30 min to block the endogenous peroxidase activity, followed by 30 min at 90°C in the same solution to recover the antigen. Immunolabeling of the *T. cruzi* antigen was performed using an antibody raised in rabbits (1∶250 dilution). The slides were then incubated with antibodies directed against *T. cruzi* for 2 h at 37°C and rinsed three times for 3 min with TBS. Next, the slides were incubated with protein A conjugated to peroxidase (1∶100) for 1 h at room temperature. The slides were washed again and imaged using 3,3-diaminobenzidine tetrahydrochloride (DAB chromogen Kit – Biocare Medical). The slides were then counterstained with Mayer's hematoxylin and mounted. Non-specific staining was controlled for by omission of the primary antisera.

### Tissue Extract Preparation for Cytokine and Nitric Oxide Measurements

The dosages of nitric oxide and TNF-α, IFN-γ and IL-10 in renal tissues were performed on days 6, 9, 12 and 18 after *T. cruzi* infection. The kidney tissues were first weighed and then immersed in equal volumes of PBS (500 µL per tissue) containing protease inhibitor (complete Protease Inhibitor Cocktail Tablets: Roche Applied Sciences, Indianapolis, IN). The protease inhibitor solution was prepared by adding one tablet to 50 mL of PBS, according to the manufacturer’s instructions. Extracts were obtained by homogenizing tissues with an electrical tissue homogenizer in the protease inhibitor buffer followed by centrifugation at 300 x *g* for 15 min, after which the supernatants were collected and stored at −70° until use. Cytokines (TNF-α, IFN-γ and IL-10) were measured according to the manufacturer’s instructions, using commercially available ELISA kits (R&D Systems, Minneapolis, MN). The cytokine concentrations were normalized, taking into account the weight of each tissue, and the results were expressed as picograms per milligram of tissue. The concentrations of nitrite/nitrate in the samples were determined by the Griess reaction after enzymatic reduction of nitrate to nitrite by using the enzyme nitrate reductase. The absorbance of the samples was measured at 570 nm using an automated microplate reader (Biorad 2550 READER EIA).

### Blood Cell Count

The cell count from the blood of uninfected and infected mice (low, medium and high load of *T. cruzi*) at 6, 9, 12 and 18 days post-infection was carried out by two different methods. To analyze the total number of leukocytes, the collected blood was mixed by inversion for five minutes, diluted 1∶20 in Türk solution and counted using a Neubauer chamber. The total number of cells was obtained by counting the four lateral quadrants and multiplying by the correction factor of the chamber and the dilution factor (total number of leukocytes x 2.5 x 20), which was expressed in mm^3^. Differential cell counts (100 cells total) were obtained using a blood smear, and the slides were stained with panoptic (Instan-prov - Neuprov ®). The cell populations were differentially counted based on the morphological features. The results were presented in absolute values (mm^3^).

### Vascular Permeability

Endothelial permeability was determined by assessing renal tissue concentration of Evans Blue (Santa Cruz Biotechnology, Heidelberg, Germany). The mice were infected with low, medium and high doses of trypomastigotes, and 9 days post-infection, the mice were anesthetized and injected with Evans Blue (30 mg/kg) in the ophthalmic plexus. After 45 min, the kidneys were perfused with 20 mL of isotonic NaCl, excised, shredded and dehydrated. Samples were homogenized in formamide, whereby the total volume was adjusted to a 20× equivalent of sample dry weight, followed by 24 h incubation at 55°C. The supernatant was separated by centrifugation at 13,000 x *g* for 30 min and Evans Blue concentration in the supernatant was quantified spectrophotometrically by measuring absorbance at 620 nm and at 740 nm to correct the contamination by heme pigments by applying a microplate reader (Biorad 2550 READER EIA). Total concentration of Evans Blue was determined from generated Evans Blue standard curve absorbance and expressed as µg/mL.

### Statistical Analysis

Statistical analysis was performed using the program "Prism" from Graphpad. Normality (Kolmogorov-Smirnov test) and homogeneous variance (Bartlett's test) were applied to all variables. When the distribution was considered normal and the variance was homogeneous, parametric tests (ANOVA with post-test of Tukey's multiple comparison) were used, and the results were expressed as the mean ± SEM. In cases when the distribution was not Gaussian, we used nonparametric tests (test "Kruskal-Wallis" with Dunn's multiple comparison) with the results expressed as median, maximum and minimum values. The Spearman nonparametric rank test was used to correlate the data [28]. The differences were considered significant when p<0.05 (5%) [29], [30].

## Results

### Effect of Different Loads of Trypomastigotes on Parasitemia and Survival Rate

**Figure 1:**
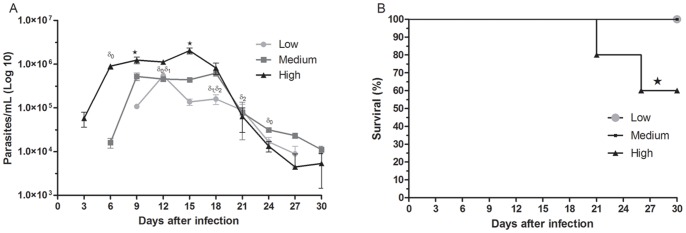
Parasitemia and survival of mice in the acute stage of *T. cruzi* infection. C57BL/6 mice were challenged with 3×10^2^ (low dose), 3×10^3^ (medium dose) or 3×10^4^ (high dose) blood trypomastigotes. Parasitemia (A) was determined by counting the number of parasites in 5 µL of blood collected from tail snips at the indicated time points. Each point represents the mean of individual values from 10 mice. In the survival curve (B), 10 animals were individually monitored for 30 days of infection. ^δ0^p≤0.05 indicates a significant difference when the mice infected with medium-inoculum were compared to the mice infected with high inoculum, ^δ1^p≤0.05 indicates a significant difference when the mice from the low-inoculum group were compared to the mice from the high-inoculum group, ^δ2^p≤0.05 indicates a significant difference when mice from the low-inoculum group were compared to mice from the medium-inoculum group, and *p≤0.05 indicates a significant difference when animals from the infected groups were compared to the uninfected control mice.

We evaluated the development of *T. cruzi* parasitemia in C57BL/6 wild type mice inoculated subcutaneously with low (300), medium (3,000) or high doses (30,000) of *T. cruzi* trypomastigotes. As shown in Figure 1A, high, medium and low parasite loads induced parasitemia that could be first detected at days 3, 6 and 9 of infection, respectively. The peak of parasitemia in mice inoculated with low and medium parasite loads was at days 12 and 9, respectively, and they did not display differences in magnitude of infection. For the mice that received high parasite loads, the peak was at day 15, which was statistically different than the other two parasite loads (p<0.05). The magnitude of infection in highly infected mice was greater at nearly all days post-infection when compared with mice challenged with low and medium inocula. Moreover, mice infected with medium loads also presented parasitemia that was statistically different (p<0.05) from mice infected with a low level of parasites at days 9 and 18 post-infection. The parasitemia of mice inoculated with low parasite load immediately dropped after reaching the peak level, while those mice that received the medium and high inocula decreased significantly after day 18 of infection. Animals infected with low or medium loads of trypomastigotes survived throughout the period of the experiment, while mice infected with high parasite loads showed a mortality of approximately 30%, with the animals dying beginning at 21 days post-infection (Figure 1B).

### Effect of Parasite Load on Urinary Excretion and Kidney Weight

**Figure 2:**
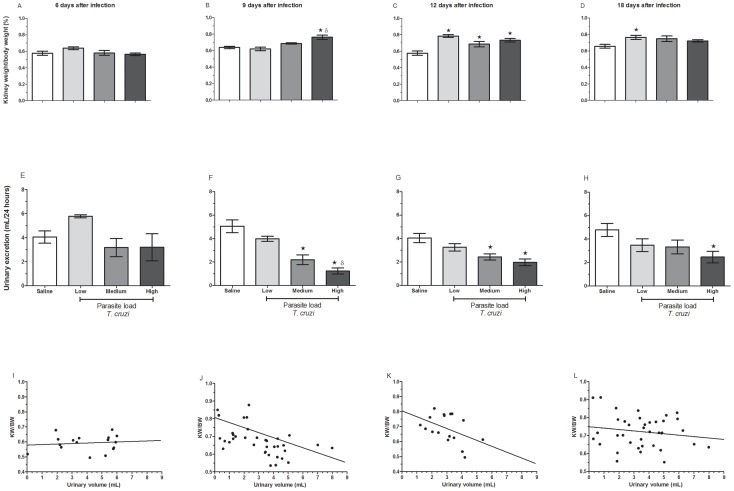
Determination of the urine excretion (24 hours) and the index between the kidney and body weight. The index between the kidney and body weight (A–D), urine excretion (E–H) and the correlation between the index and urine excretion (I–L) were evaluated as indicators of renal lesions. The bodies and kidneys of infected and uninfected mice were weighed at the indicated time points (6, 9, 12 and 18 days p.i.) to calculate the index. At the same time points, the animals were placed in metabolic cages for 24 hours to quantify the urine volume. *p≤0.05 indicates a significant difference between the animals that received a high inoculum and the uninfected animals. ^δ^p≤0.05 indicates a significant difference between the animals that received a high inoculum and the animals that received the low inoculum.

To investigate whether differences in parasite load could affect kidney injury, the functional activity of this organ was addressed in mice during the acute phase of infection (at 6, 9, 12 and 18 days post-infection). On day 6 post-infection, no significant differences in the index between the kidney weight (KW) and body weight (BW) were observed (Figure 2A). As seen in Figure 2B, there was an initial variation in the renal weight coefficient between the kidneys of the infected and non-infected groups at 9 days post-infection. In addition, the difference (p<0.05) was parasite load-dependent because only mice infected with the highest inoculum (3×10^4^ parasites) had higher renal weight coefficients than those infected with the low parasite inoculum. At 12 days after infection, there was an increase in this index (p<0.05) in all infected groups (Figure 2C). When we analyzed the different groups at 18 days post-infection, we observed renal compensation in the groups infected with the medium and high doses, while those infected with the lowest inoculum displayed an increase in coefficients compared to controls (Figure 2D). It is worth noting that the increase in the index of kidney weight to body weight was due to an increase in the weight of kidneys because no significant changes in body weight between the different groups was observed (data not shown).

The urine excretion in the infected groups over a 24-hour/period robustly started to decrease at 9 days post-infection (Figure 2E–H). In comparison with the uninfected animals, the low-dose group showed a slight reduction, but the groups infected with medium and high inocula at days 9 (Figure 2F) and 12 (Figure 2G), or only those infected with high inocula at day 18 (Figure 2G), had a more pronounced and significant (p<0.05) reduction in urinary excretion. The volume of urine from the different groups of animals remained unchanged on the sixth day of infection (Figure 2E). Based on these results, there was a negative correlation (p<0.05 and Rho = −0.6) between the renal coefficient and the volume of urine excretion beginning on day 9 of infection, and this correlation was dependent on the parasite load (Figure 2J). Overall, the degree of the reduction in urinary excretion was inversely proportional to the renal/body weight coefficient.

### Effect of Parasite Load on Renal Biochemical Parameters of Mice Acutely Infected with *T. cruzi*

**Figure 3:**
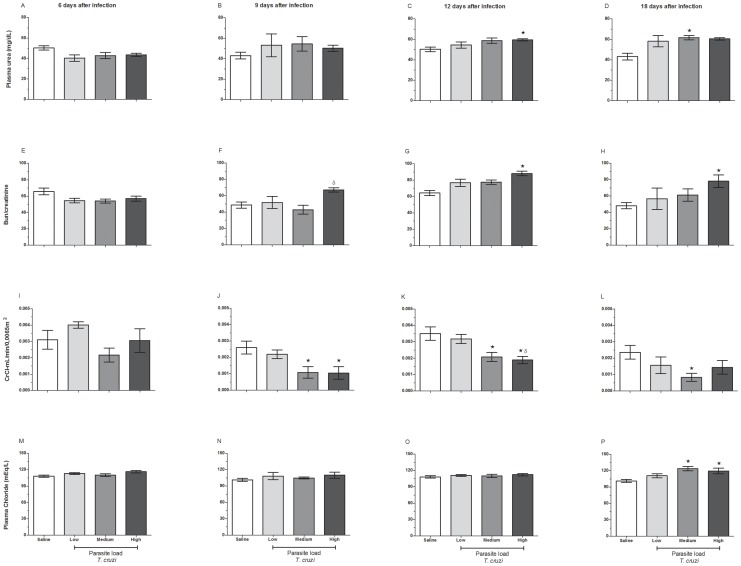
Effect of *T. cruzi* parasite loads on plasma urea concentration, BUN/creatinine ratio, creatinine clearance and plasma chloride ion levels. C57BL/6 mice were challenged with 3×10^2^ (low dose), 3×10^3^ (medium dose) or 3×10^4^ (high dose) blood trypomastigotes, and 6, 9, 12 and 18 days post-infection, the plasma and urine (24 hours) of these animals were collected. The plasma urea (A–D) and creatinine levels were measured, and the ratios between blood urea nitrogen (BUN) and creatinine (E–H) were calculated. To determine the creatinine clearance, the urine creatinine levels were measured over a 24-hour period (I–L). The concentration of chloride ions (mEq/L) was measured in the plasma from the same mice (M–P). We used commercial kits for these analyses, as described in Materials and Methods. Each bar represents the mean ± standard deviation of individual values from 10 mice. *p≤0.05 indicates a significant difference when animals from the highly infected group were compared to the uninfected control animals.

Serum levels of urea and the relationship between the levels of blood urea nitrogen (BUN) and serum creatinine were measured as standard indicators of renal function. After 6 and 9 days of infection, we observed that the differences in the plasma urea between the groups remained insignificant despite a tendency towards an increase at day 9 (Figure 3A–B). On day 12, the mice infected with high parasite loads showed a significant increase in the plasma urea when compared with uninfected controls (Figure 3C). After 18 days of infection, we detected a significant elevation (p<0.05) in the serum levels of urea, but only in the mice infected with a medium parasite load (Figure 3D). When we evaluated the relationship between the levels of blood urea nitrogen (BUN) and serum creatinine, we noted that results were quite similar to those regarding the serum levels of urea. Overall, no significant difference at 6 and 9 days post-infection (Figure 3E–F) was observed; however, the animals infected with the high parasite loads displayed a significant increase (p<0.05) in this ratio at 12 and 18 days post-infection when compared to uninfected controls (Figure 3G–H).

After evaluating the coefficient and quantifying urinary excretion, we indirectly evaluated the glomerular filtration capacity by determining creatinine clearance in the mice infected with low, medium and high doses of parasites. As depicted in Figure 3I, no significant differences in creatinine clearance were observed in mice infected with different loads of trypomastigotes on 6 days after infection. In contrast, there was a parasite load-dependent reduction (p<0.05) in creatinine clearance in the infected animals at 9 and 12 days post-infection (Figure 3J–K), mainly in the groups inoculated with medium and high doses of parasites. Although a similar trend in the reduction of the creatinine clearance was observed at day 18, a statistically significant difference (p<0.05) was found only in mice infected with the medium inoculum (Figure 3L).

Evaluation of the chloride ion levels in the plasma is an important biochemical parameter for diagnosing tubular disorders of the kidney. Although our results demonstrated only a small and insignificant elevation in the levels of the chloride ion at 6, 9 and 12 days post-infection, the animals infected with medium and high inocula of parasites displayed an increased retention of the plasma chloride ion at 18 days post-infection compared to controls (p<0.05) (Figures 3M–P).

### Effect of Parasite Load on the Presence of *T. cruzi* Amastigotes in Kidney Tissues

**Figure 4:**
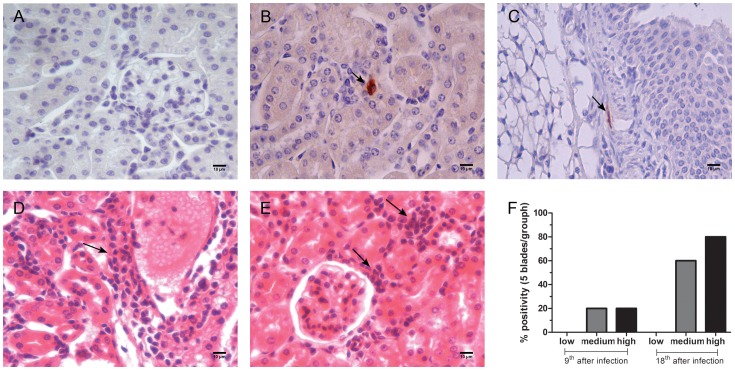
Analysis of the presence of *T.cruzi* amastigotes and inflammatory infiltrates in the renal tissues. C57BL/6 mice were challenged with low, medium and high loads of trypomastigotes, and at 9 and 18 days post-infection, the inflammatory infiltrate and the presence and location of *T. cruzi* amastigotes in the renal tissues were evaluated. *T. cruzi* amastigotes were found in both cortical/medullary (A) and peri-renal (B) tissues. The inflammatory infiltrate was evidenced in the tubular region (C) and in the Bowman’s capsule (D). After demonstrating the presence of nests of *T. cruzi* amastigotes and the inflammatory infiltrates, we evaluated the comparative percentage of positive antigen labeling for *T. cruzi* in 5 different slides collected from the different inocula at 9 and 18 days post-infection (E).

After determining that parasite load was associated with the degree of renal damage, we investigated whether infection could induce the inflammatory infiltrate and the presence and localization of *T. cruzi* amastigotes in the renal tissues. By comparing the infected with the uninfected mice, we identified nests of amastigotes in the cortical/medullary regions (Figure 4B) and in the peri-renal regions (Figure 4C) only in mice infected with the medium and high inocula of parasites (Figure 4A–C). The presence of these nests was accompanied by a slight increase in the inflammatory infiltrate of mononuclear cells in the tubular regions (Figure 4D) and in the Bowman capsules around the glomeruli (Figure 4E).

The antigen labeling for amastigotes was observed in 20% and more than 50% (60 to 80%) of the slides analyzed in the groups infected with the medium and high doses of *T. cruzi* after 9 and 18 days of infection, respectively (Figure 4F). To our surprise, we did not observe antigen labeling in the slides of the group infected with the lowest doses of *T. cruzi* (Figure 4F).

### Evaluation of the Effects of the Parasite Load on the Renal Histopathological Damage Caused by Acute *T. cruzi* Infection

To evaluate the histological structure of the kidneys in infected mice, we also measured the numerical density of the glomeruli, the volume of the glomeruli and the volume of the kidney. According to our results, challenge with low, medium and high inocula of blood trypomastigotes did not alter the numerical density of the glomeruli or the glomeruli volume at 9 or 18 days post-infection (data not shown).

### Effect of Parasite Load on the Increase in Immune Cells during Acute *T. cruzi* Infection

**Figure 5:**
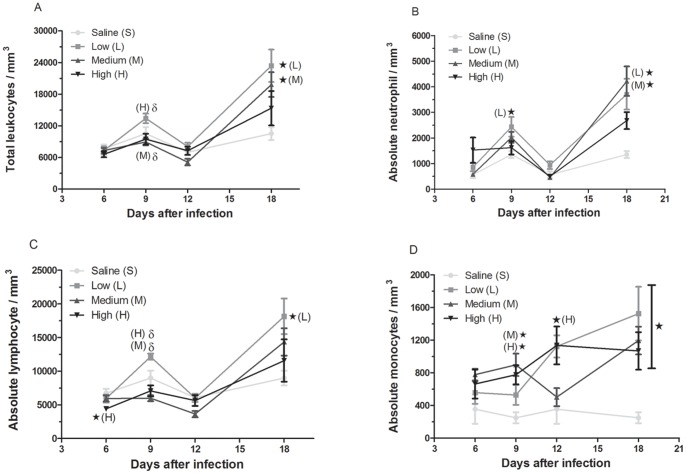
Increased circulating cells in mice infected with *T. cruzi*. C57BL/6 mice were infected with increasing doses of trypomastigotes, and at 6, 9, 12 and 18 days post-infection the number of cells/mm^3^ in the blood was determined. At each time point, the total leukocytes (A), neutrophils (B), lymphocytes (C), and monocytes (D) were measured. Total cells were counted using a Neubauer chamber, and the differential cell counts (100 cells total) were obtained using stained blood smear slides. The data are reported as the means ± SEM of 10 mice. *p<0.05 versus the uninfected group.

Acute kidney injury, such as that caused by ischemia/reperfusion, may induce an increase in the number of circulating immune cells, including lymphocytes and neutrophils [31]–[33]. Previous results have also demonstrated that *T. cruzi* infection in BALB/c mice induced acute renal ischemic/reperfusion lesions [16]. Thus, we evaluated the influence of parasite load on the blood immune cell populations during acute *T. cruzi* infection (Figure 5). In general terms, the number of leukocytes and their subpopulations were significantly altered in the blood samples from infected animals depending on the period of infection (Figure 5). On the sixth day of infection, there was only a significant decrease in the number of circulating lymphocytes in the mice infected with high parasite loads (Figure 5C). At day 9, the number of neutrophils was altered by the low-dose infection and the number of monocytes was altered by the medium and high doses (Figure 5, B and D). On the twelfth day, there was a significant increase (p<0.05) in monocyte numbers in mice infected with high parasite loads (Figure 5D). At 18 days post-infection, the total number of leukocytes was increased (p<0.05) in the animals infected with low and medium doses (Figure 5A). Moreover, the low and medium doses of parasites induced a significant increase (p<0.05) in the total number of neutrophils, and all of the inocula induced an increase (p<0.05) in the number of monocytes (Figure 5, B and D). As a control, we noted that the number of cells from the uninfected mice remained unaltered at both time points.

### Effect of Parasite Load on the Nitric Oxide (NO) and Cytokine Production in Kidney Tissues after Acute *T. cruzi* Infection

**Figure 6:**
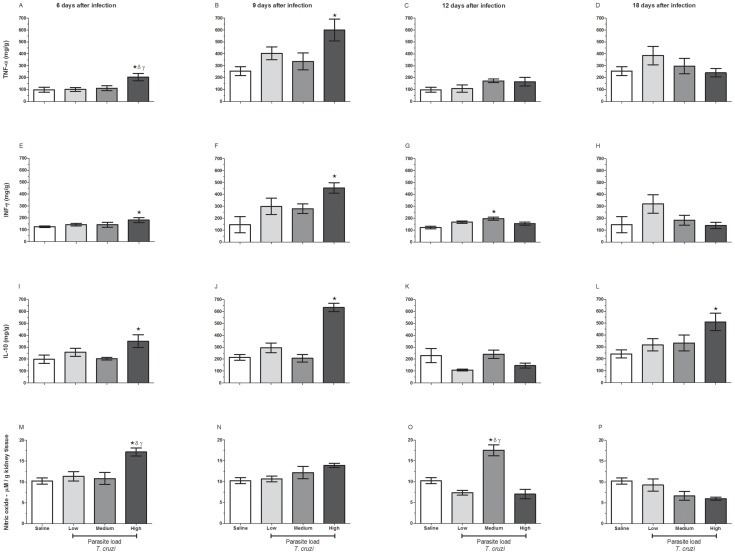
Effect of *T. cruzi* parasite loads on cytokine and nitric oxide production in kidney tissues. C57BL/6 mice were challenged with low, medium and high loads of blood trypomastigotes. At 6, 9, 12 and 18 days post-infection they were euthanized and their kidneys were removed to measure the concentrations of cytokines and nitric oxide. The cytokines TNF-α (A–D), IFN-γ (E–H) and IL-10 (I–L) were measured according to the manufacturer’s instructions, using commercially available ELISA kits. For measurement of nitric oxide, the Griess reaction was used. The absorbance was read at 570 nm. *p≤0.05 indicates a significant difference when animals from the medium and highly infected groups were compared to the uninfected control mice.

On days 6 and 9 post-infection, only mice infected with high doses of *T. cruzi* had a significant increase in the production of the proinflammatory cytokines TNF-α (Figure 6A–B) and IFN-γ (Figure 6E–H). The production of both cytokines was not sustained after 9 days (Figure 6C–D and 6 G–H) because only animals infected with medium doses of parasites showed a significant increase in IFN-γ at 12 days after infection. The production of the anti-inflammatory cytokine IL-10 was enhanced in animals infected with high doses of the parasite, and this increase occurred on all days after infection except on day 12 (Figure 6I–L). We observed that at 6 days after infection, there was a significant increase in NO production in the mice infected with high doses of the parasite (Figure 6M). This increase was not sustained on other evaluated dates, except in mice infected with the medium dose of the parasite, which produced high NO levels at 12 days after infection (Figure 6N–P).

### Effect of *T. cruzi* Parasite Load on Vascular Permeability in Kidney Tissues

**Figure 7:**
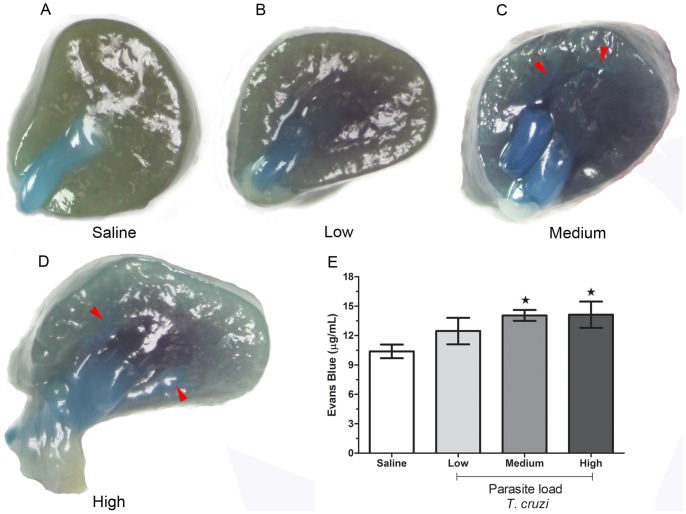
Effect of *T. cruzi* parasite loads on vascular permeability in the kidney tissue. C57BL/6 mice were challenged with low, medium and high loads of trypomastigotes and at 9 day post-infection, the accumulation of Evans Blue in the renal tissues was assessed. In A–D, a representative image of Evans Blue accumulation in the kidney from each group is demonstrated. E shows the mean percentage ± SEM of Evans Blue accumulation in the renal parenchyma. *p≤0.05 indicates a significant difference when mice from the medium and highly infected groups were compared to the uninfected control mice.

Knowing that the attraction and transmigration of immune cells is a process associated with increased vascular permeability, we evaluated renal permeability in mice infected with different doses of *T.cruzi*. Our results showed that vascular permeability was affected in a parasite load-dependent manner (Figure 7). As depicted in Figure 7A, uninfected animals had a small accumulation of Evans Blue in renal tissues. The accumulation of Evans Blue was higher in the mice infected with higher doses of the parasite (Figure 7C–D, red arrows). The kidneys of mice infected with medium and high doses of the parasite exhibited increased accumulation of Evans Blue compared with uninfected mice (Figure 7E).

## Discussion

In this report, we demonstrate that the kidney is a target of damage during experimental acute *T. cruzi* infection and that the status of this injury and the resulting impaired renal function are more evident in mice that have been infected with high parasite loads. In our experiments, mice acutely infected with *T. cruzi* demonstrated a significant increase in the renal inflammatory infiltrate, renal vascular permeability, the coefficient between kidney weight and body weight, plasma chloride ion levels and the relationship between the levels of blood urea nitrogen and serum creatinine. In addition, nitric oxide and cytokine (TNF-α, IFN-γ and IL-10) production in renal tissues was also augmented. Furthermore, we also observed a decrease in urinary excretion and in creatinine clearance, mainly in the mice infected with the highest parasite loads.

First, we demonstrate that the logarithmic concentration of parasites affected the development of parasitemia and the mortality rate. The differences in the intensity of parasitemia between the differentially infected mice were found at the onset, the peak of infection and the time at which the infection started to decrease. In addition, only mice infected with high parasite load had a mortality rate, which was approximately 30%. The lack of correlation between the onset and peak of parasitaemia between the groups could be explained by the sensitivity of the method applied for the detection of the parasite [25] and the biological cycle of *T. cruzi* since the binary division of the parasite in infected phagocytic cells favors the appearance of blood trypomastigotes firstly in mice inoculated with high doses. Additionally, the host immune response will be more competent in containing the spread of the parasite in infected animals at the lowest dose, which explains the decline of blood forms on this group after the twelfth day of infection. Our parasitemia results are in agreement with the literature, which reports that the beginning of the parasitemia and the progress of acute infection in mice may vary depending on the parasite load [18], [34]–[35]. Similar to our findings, it was also demonstrated that approximately 30 days post-infection, few parasitic forms remain [34]. Regarding mortality, it is possible to suggest that this variable is partially dependent on the parasite load because some mice infected with the higher inoculum died from the infection. Notably, mortality during *T. cruzi* infection is also dependent on the balance in the production of reactive nitrogen intermediates such as NO and pro-inflammatory and anti-inflammatory cytokines such as TNF-α, INF-γ and IL-10 [36]–[37]. As demonstrated in this work, the balance in the production of these molecules was much more deregulated in mice infected with high parasite loads.

The kidneys’ ability to perform their physiological functions, such as glomerular filtration and renal tubular reabsorption, may be monitored through assessment of many biochemical parameters including the plasma ions, serum and urinary metabolites. Such monitoring has already been performed either in experimental models or in patients who received kidneys from donors infected with *T. cruzi* [15], [38]–[41]. In our experimental model, we observed these physiological changes, but we also demonstrated that these effects are correlated with the concentration of the *T. cruzi* inoculum because renal functional abnormalities occurred mainly in mice that received the medium and high inocula. Concerning these findings, it is well established that during the acute phase of Chagas disease, most of the cases of renal injury are due to cardiac hemodynamic alterations (e.g., cardiac output and blood pressure); however, a recent study by Oliveira and collaborators (2009) suggested that acute renal injury may occur in the absence of these cardiac hemodynamic alterations [17]. In this work, they demonstrated that Fas-L knockout mice infected with *T. cruzi* presented a severe kidney injury characterized by very early glomerular deposition of IgM, intense renal inflammatory response, premature death and absence of severe myocarditis. Our findings, which demonstrate the differential presence of amastigote nests, inflammatory infiltrates and alterations of biochemical parameters during the early days of infection of the highest infected mice, reinforce this hypothesis. Thus, IgM deposition would promote the formation of immune complexes that result in premature glomerulopathy [17], [42] and the alteration of renal function. Together, the increased inflammatory process demonstrated here, would promote cellular injury and dysregulated kidney protection. It is also important to report that kidney injury has also been observed after experimental acute infection with the protozoa *Leishmania*, a trypanosomatid with a close phylogenetic relationship with *T. cruzi*. During *Leishmania* infection, in addition to changes in the biochemical parameters related to kidney injury, the renal damage is also characterized by intense plasma cell exudate, albuminous degeneration of the tubules, proliferation of mesangial cells followed by thickening of the Bowman’s capsule, hyalinization, glomerular sclerosis and the presence of hyaline casts in the loop of Henle [43]–[46].

Overall, the pathological effects of the highest parasite loads on kidney injury, such as the increase in the index of kidney/body weight, production of TNF-α, IFN-γ, IL-10 and NO and the decrease in the urinary excretion are more evident in the early days of acute infection. These events are most likely due to an early reaction of mice exposed to high parasite loads. As the kidney is capable of self-regulation in the presence of an injury, the mice that first presented this dysregulation will develop a compensatory adjustment in these parameters earlier compared with the other groups, which we can see on day 18 after infection. This compensatory activity that promotes the rearrangement of the kidney tissue is already well known and is attributed to several factors, such as structural enlargement, evidenced by a hypertrophy of the glomerulus [47], or by tubuloglomerular feedback and the activities of angiotensin and prostaglandins, as demonstrated by Schnermannet *et al* [48].

In the present work we also show that pro-inflammatory molecules are induced in the kidney during experimental *T. cruzi* infection. An increase in the inoculum used was correlated with the increased concentration of the pro-inflammatory cytokines TNF-α and IFN-γ. To our surprise, renal tissues from mice infected with medium or high doses of the parasite also demonstrated an increased production of the anti-inflammatory cytokine IL-10. Our results are in agreement with the literature because several studies using acute experimental models of infection or in humans have reported the critical role of TNF-α [49], [51], IFN-γ [52] and IL-10 [53], [54] in modulating the immune response in the acute phase of the Chagas disease. Concerning IL-10, it is important to note that elevated IL-10 production is associated with control of *T. cruzi* and protection from fatal acute myocarditis [55], [56], a condition that is much more evident in mice infected with high parasitic loads. In addition, NO was also increased. It is known that reactive nitrogen intermediates such as NO have been described as essential molecules for defense against *T. cruzi* during the acute stage of the infection [57]–[59]. In our experiments, NO production was more evident in mice infected with higher doses of the parasite and was particularly pronounced in the sixth day of infection, suggesting that the production of this intermediate is related to parasite load and occurs during the earlier days of infection. Our results agree with findings in the literature showing that, *in vitro*, the production of these pro-inflammatory cytokines and nitric oxide by renal cells may be associated with renal lesions and inflammation during *T. cruzi* infection [12].

Concerning the inflammatory processes of the kidney, we demonstrate that despite the lack of morphometric variations in the kidney, the animals infected with *T. cruzi* had a significant inflammatory infiltrate with a predominance of mononuclear cells in the tubular region and in the Bowman’s capsule around the glomeruli. The inflammatory infiltrate was accompanied by the deposition of the amastigote form of the parasite and an increase in the absolute numbers of total leukocytes, lymphocytes and monocytes in the blood. The findings of renal inflammation were expected because we demonstrated that renal vascular permeability was most evident in the mice receiving the highest doses of the inocula. It is important to note that the increase in vascular permeability could also explain the temporary increase in the kidney weight because a hemodynamic imbalance may lead to edema, one of the most important signs of Chagas disease [60]. Regarding the deposition of amastigotes in renal tissues our findings are in contrast to the results of Oliveira *et al* 2009 [16]–[17] since they do not associate the presence of parasites in the renal parenchyma with the kidney injury of mice infected with *T. cruzi*. We believe this disagreement was observed because of two factors: the different strains of mice tested (BALB/c) and the number of trypomastigotes inoculated (1000 forms), a value considerably lower than used here to the highest inocula.

Taken together, our results demonstrate in an experimental model of acute *T. cruzi* infection that different parasite loads differentially affected the kidney biology by impairing kidney function and inducing a renal inflammatory process. Moreover, we demonstrated that the deposition of *T. cruzi* amastigotes in renal tissues was dependent on the number of parasites inoculated. Thus, we have summarized the kidney involvement during acute *T. cruzi* infection in mice infected with different parasite loads. These findings emphasize the evidence concerning renal pathology. This may be important for better understanding of the pan-infectivity of parasites and consequently the understanding of disease progression in experimental infection models.
